# Fertility Intentions and Their Realization in Couples: How the Division of Household Chores Matters

**DOI:** 10.1177/0192513X19848794

**Published:** 2019-05-15

**Authors:** Bernhard Riederer, Isabella Buber-Ennser, Zuzanna Brzozowska

**Affiliations:** 1 Austrian Academy of Sciences, Vienna, Austria; 2 University of Vienna, Vienna, Austria

**Keywords:** fertility intentions, division of household labor, realization of intentions, family work, Generations and Gender Survey

## Abstract

Most research on Europe indicates that a gender-balanced division of family work tends to increase childbearing probabilities, but empirical results vary substantially. The present article proposes explanations for this observed discrepancy. It develops prior research further by (1) studying short-term fertility intentions and their realization within the subsequent 4 years, (2) analyzing the role of the spouses’ satisfaction with the division for the effects that the division may have on childbearing, (3) proving a mediation by relationship satisfaction, and (4) considering gender as well as parity as moderators. Using data from two waves of the Generations and Gender Survey, we show that the division of work affects childbearing intentions. We find that the effect (a) depends on the spouses’ satisfaction with the division, (b) is partly moderated by relationship satisfaction, and (c) varies by parity. The division of household labor, however, seems of less importance for the realization of childbearing intentions.

During the past decades, fertility intentions have gained importance in family research, being analyzed from different perspectives and in different national contexts (e.g., Hagewen & Morgan, 2005; Liefbroer, 2009; Sobotka, 2009). Prior research has shown the importance of partnership for childbearing intentions and confirmed, in particular, the role of partnership stability (Qu, Weston, & Kilmartin, 2000), agreement with the partner regarding childbearing intentions (Testa, Cavalli, & Rosina, 2014), or relationship satisfaction in general (Berninger, Weiß, & Wagner, 2011). In addition, scholars have argued that women’s participation in the labor market and men’s involvement in unpaid family work affect the decision for childbearing (Goldscheider, Bernhardt, & Lappegård, 2015; Neyer, Lappegård, & Vignoli, 2013). Correspondingly, numerous studies have shown links between the division of family work and individual childbearing (e.g., Cooke, 2009; Dommermuth, Hohmann-Marriott, & Lappegård, 2017; Mencarini, Vignoli, & Gottard, 2015).

Most studies indicate that an equal distribution of labor between partners increases fertility. Nevertheless, studies reveal substantial variations in the link between a couple’s division of family work (household chores, childcare, etc.) and childbearing indicators (childbearing intentions, childbearing probabilities, etc.): Even in one and the same study, both an equal and an unequal division are occasionally associated with higher childbearing probabilities (e.g., Miller Torr & Short, 2004). To our knowledge, this variation in the link between family work and fertility has rarely been explored systematically so far (for a notable exception see Mills, Mencarini, Tanturri, & Begall, 2008). The present article aims to fill this research gap. Using two-wave panel data from four European countries, we address mechanisms that potentially affect the link between the division of family work and (further) childbearing. In particular, we argue that the effects of the division of family work on childbearing are dependent on a couple’s satisfaction with this division, on gender, and on parity. While previous research has focused on childbearing intentions or probabilities only, we study the effects of the division of household chores on fertility intentions as well as on their realization.

## Family Work and Childbearing

### Existing Empirical Evidence

The link between family work and childbearing receives considerable attention in the literature. Most existing research focuses on births, that is, childbearing probabilities (e.g., Brodmann, Esping-Andersen, & Güell, 2007; Cooke, 2004, 2009; Mencarini & Tanturri, 2004; Miller Torr & Short, 2004; Oláh, 2003; Thomson, 1997). Other studies analyze childbearing intentions (e.g., Iwinska-Nowak, 2011; Mencarini et al., 2015; Mills et al., 2008; Tazi-Preve, Bichlbauer, & Goujon, 2004). The literature usually concludes that a balanced division between partners increases the intention as well as the actual likelihood of having (additional) children (e.g., Dommermuth et al., 2017; Mills et al., 2008). Nevertheless, a closer look at existing evidence suggests that results are far from being conclusive. While most studies indeed indicate a positive effect of male contributions to family work on childbearing (e.g., Mills et al., 2008), others document a negative effect: A recent study from Sweden, for instance, revealed that egalitarian men are characterized by higher levels of childlessness (Bernhardt, Goldscheider, & Turunen, 2016). Others report mixed results (e.g., Cooke, 2009, on childbearing probabilities; Tazi-Preve et al., 2004, on childbearing intentions) or do not find any significant effects (e.g., Brodmann et al., 2007). Most interestingly in this respect, Miller Torr and Short (2004) showed that both modern and traditional couples are characterized by higher probabilities of childbearing. In addition, using macro indicators, Mills (2010) found evidence that lower as well as higher gender equality in a relationship can be linked to lower fertility intentions.

Studies offering an explanation for the variation in the link between the division of family work and childbearing indicators are rare. To our knowledge, only two studies tested whether the effect of the division of family work varies with contextual circumstances. Brodmann et al. (2007) argued that male participation in household work reduces the motherhood penalty for women and thus increases the probability of childbearing. They analyzed whether this link is dependent on female bargaining power and obtained inconclusive results. Mills et al. (2008) assumed that the effects of male participation on childbearing intentions are conditional on role strain and women’s double burden. They argued that intentions of women are only reduced by taking on a large share of the household labor if other burdens are already high (e.g., due to childrearing). Results showed that mothers doing more than three quarters of housework had lower childbearing intentions than childless women engaged in less housework (Mills et al., 2008). Moreover, Goldscheider, Bernhardt, and Brandén (2013) linked gender role attitudes prior to childbearing with later sharing of family work in Sweden. They found that an inconsistency between a woman’s ideals (before birth) and reality (after birth) significantly delayed continued childbearing. The present article will address further factors that may influence the effect of the division of family work on childbearing.

Against this background, several aspects need to be addressed. First, authors use different kinds of operationalization to measure family work and gender equity, which partly explains the ambiguous results. Indicators include attitudes (e.g., Thomson, 1997), weekly hours of family work (e.g., Brodmann et al., 2007), the division of tasks between partners (e.g., Cooke, 2004, 2009), or satisfaction with the division of these tasks (e.g., Tazi-Preve et al., 2004). Second, many studies are restricted to women (e.g., Mencarini et al., 2015; Mills et al., 2008), thereby disregarding potential gender differences. Although existing evidence is not fully consistent, findings clearly indicate that gender differences may matter (Iwinska-Nowak, 2011; Tazi-Preve et al., 2004). Third, most studies focus on parents with one child: on their intentions to have further children or their actual transition to the second child (e.g., Brodmann et al., 2007; Cooke, 2004, 2009; Miller Torr & Short, 2004; Oláh, 2003). Mencarini and Tanturri (2004), however, report different results for the transition to the second and the third child, respectively. As Cooke (2004) notes, consequences of the division of family work may depend on the presence of children. More detailed analyses by parity are needed to shed further light on the complex issue of gender equity and fertility (Goldscheider et al., 2013; Mills, 2010).

### Theoretical Perspectives

Scholars addressing fertility intentions and their realization mainly refer to the theory of planned behavior (TPB; Ajzen, 1991). According to the TPB, intentions depend on attitudes, social norms, perceived behavioral control, and background factors. In addition, enablers and restrictions are relevant for realizing intentions. The division of work within the family might be regarded as a background factor that affects the formation of fertility intentions and as an enabler facilitating the realization of existing intentions. As the TPB does not specify how the division of work affects intentions and their realization, we discuss alternative theoretical frameworks that explicitly account for this aspect.

In the previous literature on this topic, arguments can be found for both positive and negative effects of the division of family work on childbearing. *Household economics theory* (Becker, 1985) argues that a clear division of tasks produces the advantage of specialization. According to this theory, utility is maximized if women and men specialize in either employment or household chores. As women have a comparative advantage with regard to “producing children,” utility is higher among couples following traditional gender role models (i.e., the man being the main breadwinner, the woman being the chief homemaker). Accordingly, childbearing is assumed to be higher among couples with traditional gender role models. Similar conclusions follow from cultural explanations: *Modernist theories* (e.g., Lesthaeghe, 2014) argue that women pursuing a professional career will postpone childbearing or forgo prior family plans. Couples following traditional role models should thus have higher rates of realization.

In contrast, a *gender-egalitarian approach* regards male contributions to family work to be a prerequisite for a recovery of fertility in contemporary European societies (Esping-Andersen & Billari, 2015; Goldscheider et al., 2015). In short, an equal division of family work takes away the double burden from women, raises relationship quality, and thus increases childbearing intentions, resulting in higher childbearing in egalitarian couples.^1^

In these prominent theories, arguments can be found for both positive and negative effects of (un)equal household labor divisions. Each of the presented theoretical perspectives, however, argues in favor of a particular direction of the association under study rather than explaining the observable variation. As a next step, we hence discuss further theoretical reflections to identify the circumstances under which traditional or modern divisions support or hinder childbearing.

### Hypotheses

When reviewing the empirical literature, we identified several shortcomings regarding the assessment of family work, as well as the consideration of gender and parity. These shortcomings guide our further elaboration of potential reasons for varying effects of the division of family work on fertility intentions and their realization.

#### Division of Family Work and Its Assessment

The well-known *Thomas Theorem* (Thomas & Thomas, 1928) emphasizes that the subjective interpretation of a situation and not the situation per se guides human action. In the same vein, psychological research suggests that an individual’s perception of the division of family work is more relevant for the consequences of an unequal division than the division itself (Coltrane, 2000; Mikula, Riederer, & Bodi, 2008). An unequal division may be perceived as fair because of an individual’s desires and values, references taken for comparison, or adopted justifications (cf. Major, 1993; Thompson, 1991). In addition, authors such as Nock (1987) and Hakim (2003) argued that differences in lifestyle preferences matter significantly and that an unequal division of family work may be accepted more readily by traditional women (and men).

Against this background, the present research differentiates between the *division of family work* itself and the individual’s *satisfaction with this division*. We hypothesize that the effect of an unequal division on fertility intentions is dependent on the satisfaction with the division (Hypothesis 1). On the one hand, an unequal division of family work is hypothesized to reduce childbearing intentions if the individual is dissatisfied with this situation (Hypothesis 1a) (cf. *gender-egalitarian approach*). On the other hand, individuals holding traditional family attitudes may be both highly content with an unequal division of tasks and have a preference for a high number of children (Hypothesis 1b) (cf. *household economics*).

In addition, we assume that relationship satisfaction mediates the effect of the division of household chores (Hypothesis 2). A more equal division of family work may increase childbearing intentions *because* it raises relationship satisfaction (cf. *gender-egalitarian approach*). Therefore, the effects of the division itself will be different when indicators of relationship quality are included in regression models. The omission of this aspect in previous research could be a contributing factor to the aforementioned inconsistent findings.

#### Varying Effects by Gender

Despite increasing participation of men in family work during the past decades, there is still a considerable imbalance in the division between spouses to the disadvantage of women (Kan, Sullivan, & Gershuny, 2011; Öun, 2013; Sullivan, Billari, & Altintas 2014). Although inequity goes along with impaired satisfaction with both underbenefited and overbenefited partners, equity theory and available empirical evidence suggest that unjust situations are more relevant to disadvantaged partners than to the advantaged (e.g., Buunk & VanYperen, 1991). Perceptions of the division of work can differ between women and men—and thus also consequences of relationship satisfaction (Mikula, Riederer, & Bodi, 2012; Ruppanner, Brandén, & Turunen, 2018). We hypothesize that a dissatisfaction with a traditional division of tasks reduces the fertility intentions of women more often than those of men (Hypothesis 3).

#### Varying Effects by Parity

Following previous studies (Goldscheider et al., 2013; Mills, 2010), we hypothesize that the effect of the division of family work varies by parity (Hypothesis 4). *Childless* persons are specific, as (a) even in egalitarian couples, the division usually becomes imbalanced after the birth of the first child (e.g., Grunow, Schulz, & Blossfeld, 2012; Twenge, Campbell, & Foster, 2003) and (b) the effects of the division of household labor can be studied without interferences of factors such as parental leave or childcare. If the division between partners reflects their attitudes (traditional or egalitarian), it will result in a higher satisfaction with the division. We hypothesize that a higher satisfaction with the division of household chores will raise childbearing intentions among childless couples (Hypothesis 4a).

Particularly for mothers, family workload increases with the birth of the first child. The division of family work is thus crucial for the transition to the second child for women (Goldscheider et al., 2013). At Parity 1, parents may want the first child to have a sibling or desire to have both a son and a daughter (Blake, 1981; Bongaarts, 2001). We hypothesize that individuals with one child who are satisfied with the division will be more likely to want a second child than those less satisfied (Hypothesis 4b). However, intentions may be stronger in egalitarian couples than in traditional couples among Parity 1 parents (Hypothesis 4c), as the burden added by the first child has been more equally distributed in egalitarian couples (cf. Mills et al., 2008).

At higher parities (i.e., 2+), the widespread two-child family ideal (Sobotka & Beaujouan, 2014) is already attained. It becomes increasingly difficult for both partners to pursue a professional career, as reconciling motherhood with employment is more complex and interruptions of employment by leave sum up to longer periods. Parents with two or more children who want further children are likely to have (very) traditional attitudes and/or a particularly strong affinity to children. We hypothesize that household labor matters less for childbearing intentions among individuals with two and more children than in lower parity groups (Hypothesis 4d).

For the realization of short-term fertility intentions, the same basic considerations apply. In particular, an unequal division could restrict realization in egalitarian couples (dissatisfied with unequal division), whereas an equal division would be an enabler in egalitarian couples (satisfied with an equal division) (Hypothesis 5a). Following the TPB framework, the division of work as a background factor may affect childbearing primarily via fertility intentions. Accordingly, we hypothesize that the division of household work is less important for the realization of existing intentions than for the formation of fertility intentions (Hypothesis 5b).

## Data and Analytic Strategy

### Sample

The present study is based on the first two waves of the Generations and Gender Survey (GGS) conducted in Austria, Hungary, France, and Poland. Data include detailed information on family formation and fertility and make it possible to analyze both fertility intentions and the realization of short-term intentions. For our analyses, we restrict the sample to 15,168 respondents aged 20 to 45 years sharing a household with their partner. Fertility intentions are only available for the respondent, not for both partners. Additionally, excluding respondents with contradictory or missing information regarding (a) short-term fertility intentions (1,119 cases were physically not capable of having children, expecting a child at the time of the first interview, not living in a heterosexual relationship, etc.), (b) household labor or professional work (1,146 cases), or other variables of interest (82 cases), the final sample of wave 1 amounts to 12,801 persons. Longitudinal analyses on the realization of fertility intentions are restricted to *panel-respondents articulating short-term childbearing intentions in Wave 1* (2,783).^2^

### Variables

Short-term fertility intentions and their realization are the two dependent variables in our study. Main explanatory variables are the division of household labor (combined with the individual’s satisfaction with it),^3^ the division of professional work, and relationship satisfaction. We briefly describe how these variables are measured and defined in the current article (descriptive statistics are available as online supplemental material).

#### Fertility Intentions

For short-term fertility intentions at Wave 1, we distinguish between respondents who (1) intend to have a(nother) child within the following 3 years, (2) plan to have a(nother) child later, and (3) do not want to have any (further) children.

#### Realization of Fertility Intentions

Focusing on respondents *wanting a(nother) child within the following 3 years at Wave 1*, we speak of realization *if a birth occurred* within 3½ years after the time of the first interview.^4^ In the countries under study, data collection of GGS waves was conducted in intervals of 3 to 4 years. For the sake of comparability, we focus on a period of 3½ years.

#### Division of Household Labor

For measuring the division of household labor (according to the respondent’s report), we construct an index summarizing four different tasks, namely, preparing daily meals, washing the dishes, shopping for food, and vacuum cleaning. First, the original scale is transformed to indicate the share of work done by the woman (1 = “always done by the man,” 2 = “usually done by the man,” 3 = “done about equally by both partners,” 4 = “usually done by the woman,” 5 = “always done by the woman”).^5^ Second, a mean index of the four items is computed (α = .87). Third, using a threshold of 3.75, we define two groups indicating a traditional (mean index above threshold) or modernized division of labor (mean index threshold and below). In other words, a division is called “modernized” if (a) the man is contributing to all four household tasks and equally to at least one of them or (b) the man is always doing one task and additionally contributes to at least one of the remaining three tasks. This strategy is in line with previous research (e.g., Mills et al., 2008). In addition, with the threshold of 3.75, the group sizes allow for further differentiation by satisfaction, as some groups are rather small (more information is available on request). Fourth, we transform the 11-point rating scale for “satisfaction with the division,” replacing the highly skewed measure by a categorial variable distinguishing three groups of approximately equal size: “moderately satisfied or less” (0 to 7), “very satisfied” (8 and 9), and “extremely satisfied” (10).^3^ Fifth, we combine the division of household labor and satisfaction with this distribution and distinguish between (1) modernized division of labor and moderately or less satisfied, (2) modernized division of labor and very satisfied, (3) modernized division of labor and extremely satisfied, (4) traditional division of labor and moderately or less satisfied, (5) traditional division of labor and very satisfied, (6) traditional division of labor and extremely satisfied.

#### Relationship Satisfaction

Participants assessed their satisfaction with their partner on an 11-point rating scale (0-10), with higher values indicating higher levels of satisfaction. This original ordinal variable is included in the models.

#### Controls

At the individual level, various aspects have proved relevant for fertility intentions in previous studies (e.g., Régnier-Loilier & Vignoli, 2011; Spéder & Kapitány, 2009; Toulemon & Testa, 2005). In addition, contexts of childbearing vary across countries (e.g., Ferrarini, 2006; Öun, 2013). Accordingly, the following sociodemographic and economic characteristics are considered as control variables: (a) gender, (b) age, (c) partner status at Wave 1, (d) separation and repartnering between waves (only in longitudinal analyses), (e) couples’ education, (f) division of professional work, (g) financial situation, (h) attitudes toward parenthood, (i) parity, and (j) country of residence (Austria, France, Hungary, Poland). With the exception of the realization of Wave 1 intentions at Wave 2 and separation/repartnering between the waves, variables refer to Wave 1.

### Analytic Strategy and Method

Our analytic strategy comprises several steps: First, we examine the role of the division of household labor and perceived satisfaction for short-term fertility intentions. Analyses are carried out (a) separately for females and males, (b) for groups of different parities (childless, Parity 1, Parity 2 or higher), and (c) separately for women and men with different parities. Finally, based on the longitudinal sample, we provide insights into the role of the division of household labor on the realization of fertility intentions. Multivariate analyses of realization are carried out for the entire sample as well as for Parities 0, 1, and 2+ separately. We do not analyze the realization of existing intentions by gender. As both partners are needed to realize childbearing intentions through pregnancy and birth within couples, we perceive it to be a couple-level variable.^6^ To adjust for different sample sizes in the four countries, weights equalizing the national sample sizes are used.

In multinomial and binomial logistic regressions, we estimate average marginal effects. We employ hierarchical model building with a stepwise inclusion of explanatory variables. Model 1 captures the bivariate association between the division of household labor and satisfaction with it, on the one hand, and short-term fertility intentions, on the other. This basic model serves as a starting point to find out if and how the association changes when we add variables of interest. In Model 2, we add relationship satisfaction as the second explanatory variable. The KHB approach (Karlson, Holm, & Breen, 2012) is used to prove mediation by relationship satisfaction in our multinomial logistic regression models. Accounting for the problem that the variance of the underlying latent variable differs between two logistic models, it proves if adding relationship satisfaction changes the effects of the division of labor. In Model 3, we additionally include the above-mentioned control variables. We decided to add them in the final step as the division of household labor, the satisfaction with this division, and relationship satisfaction may all vary by age, partner status, or couple’s education (alternative models and respective tests for mediation are shown in the online supplemental material).

## Results

### The Division of Household Work and Childbearing Intentions: First Descriptive Results

**Figure 1.**
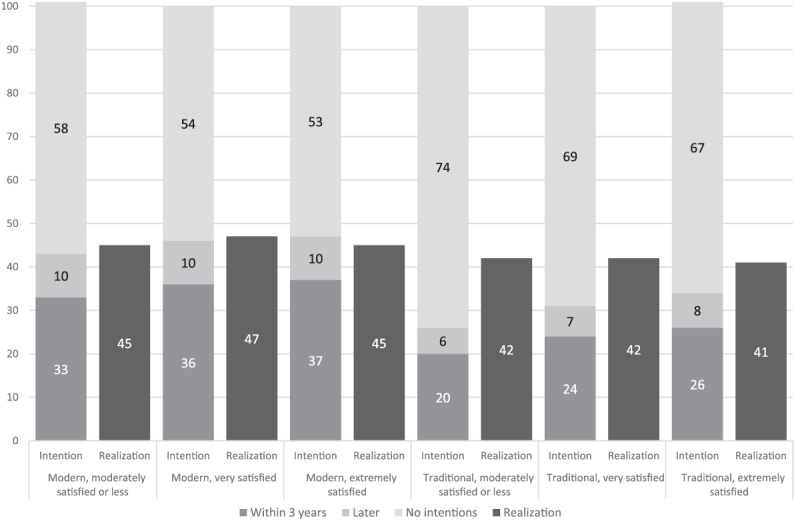
Childbearing intentions at Wave 1 and their realization at Wave 2 by division of household labor. *Note*. Source: GGS Waves 1 and 2 (own calculation; weights equalizing national sample sizes). Persons aged 20 to 45 years, cohabiting with a partner (*N* = 12,801). Realization refers to those intending to have a child within the next 3 years at Wave 1 who participated at Wave 2 only (*N* = 2,783).

Descriptive analyses (Figure 1) show that individuals with a modernized division of household chores report the intention to have a child within the next 3 years more frequently than those with a traditional division (33%-37% and 20%-26%, respectively). However, modern couples are childless (24%-27%) to a larger degree than couples with a traditional division (9%-12%). This first finding thus emphasizes the necessity of parity-specific analyses.

Among modern as well as traditional couples, short-term childbearing intentions are expressed more frequently by respondents who are content with their division of housework than by those who are dissatisfied (37% and 36% vs. 33%; 26% and 24% vs. 20%). This second finding clearly supports our approach of distinguishing between the division and its subjective perception. It furthermore raises the question to what degree the effects of the division of work on childbearing intentions are mediated by relationship satisfaction in general.

### The Division of Household Work and Childbearing Intentions of Women and Men

In multinomial regressions, childbearing intentions are analyzed separately for women and men (Table 1). The coefficients are expressed as probabilities relative to the reference category: positive (negative) numbers signify a higher (lower) probability of intending to have a child within 3 years, or wanting a child later, or not intending to have a child than those of the reference category. When not controlling for anything else (Model M1), women in couples with a modernized division of household labor report short-term childbearing intentions more often than women in couples with a traditional division. In addition, the probability of short-term childbearing intentions rises with increasing satisfaction of the division. In the first model, short-term fertility intentions are lowest among women who are not satisfied with their traditional division of household labor (see M1). Accounting for the effects of relationship satisfaction (M2), differences in intentions due to the satisfaction with the division of work disappear. A modernized division of household labor, however, still goes along with stronger childbearing intentions, demonstrating that the effect of the division of work is only partly mediated by relationship satisfaction. If controls are included in the analysis (M3), coefficients become smaller, but the effects of a modernized division remain significant. Results for men are similar to those for women. However, the division of household labor becomes insignificant in the final model (Table 1, M1-M3).^7^

**Table 1.** Effects of the Division of Household Labor on Childbearing Intentions by Gender (Average Marginal Effects).

Model	M1			M2			M3		
Childbearing intention	3 years	Later	None	3 years	Later	None	3 years	Later	None
Women (*N* = 7,586)									
Division of household labor									
Modernized, moderately satisfied, or less	.09***	.03*	−.11***	.14***	.03**	−.17***	.02	.01	−.03(^*^)
Modernized, very satisfied	.12***	.03**	−.15***	.14***	.03**	−.17***	.03*	.01	−.05**
Modernized, extremely satisfied	.14***	.02*	−.16***	.13***	.02*	−.16***	.03(^*^)	.00	−.03*
Traditional, moderately satisfied, or less	−.03*	−.01	.05**	.02	−.01	−.01	.02	.00	−.02
Traditional, very satisfied	.00	.00	.00	.02	.00	−.02	.02	.01	−.03*
Traditional, extremely satisfied (ref.)	0	0	0	0	0	0	0	0	0
Relationship satisfaction (from 0 “not satisfied” to 10 “very satisfied”)				.03***	.00	−.03***	.01***	.00	−.01***
Cragg and Uhler’s adjusted *R*^2^	.04			.05			.54		
Men (*N* = 5,215)									
Division of household labor									
Modernized, moderately satisfied, or less	.06*	.01	−.07*	.12***	.00	−.13***	.04	−.01	−.03
Modernized, very satisfied	.09***	.01	−.11***	.12***	.01	−.13***	.00	.00	.00
Modernized, extremely satisfied	.08***	.03*	−.11***	.08***	.03*	−.10***	−.01	.00	.01
Traditional, moderately satisfied, or less	−.08*	−.01	.09**	−.03	−.01	.05	−.03	−.01	.04
Traditional, very satisfied	−.02	−.02	.04	.01	−.02(^*^)	.01	.02	−.02	.00
Traditional, extremely satisfied (ref.)	0	0	0	0	0	0	0	0	0
Relationship satisfaction (from 0 “not satisfied” to 10 “very satisfied”)				.04***	.00	−.04***	.03***	−.01(^*^)	−.02***
Cragg and Uhler’s adjusted *R*^2^	.02			.03			.51		
Controls							incl.		
Constant	incl.			incl.			incl.		

*Note.* Model M3 controls for age, marriage, parity, educational homogamy, division of professional work, economic situation, attitudes toward parenthood, and country of residence. Source: Generations and Gender Survey Wave 1 (own calculation; weights equalizing national sample sizes). Persons aged 20 to 45 years cohabiting with a partner.

(*) *p* < .1. *p < .05. **p < .01. ***p < .001.

Comparing models M1 and M2 (KHB approach), tests confirm that for both women and men, adding relationship satisfaction changes the effects of the division of household labor on fertility intentions. In particular, the effect of the division on *not* wanting a (further) child seems to be mediated by relationship satisfaction (see online supplemental material). In addition, higher relationship satisfaction leads to higher childbearing intentions among both women and men. The estimated coefficient decreases in size from M2 to M3, but it remains highly significant (Table 1).

### The Division of Household Work and Childbearing Intentions by Parity

Differences between childless persons, parents with one child, and parents with two or more children are considerable (Table 2). *Childless* individuals with a modernized division of household work want to remain childless less often than those reporting a traditional division of household work (M1 to M3).^8^ Among parents with *one child*, fertility intentions are stronger with a modernized division of work unless respondents are highly satisfied with this modernized division (M1 to M3). Irrespective of whether the division of household labor is modernized or traditional, fertility intentions are less frequent among parents with *two or more children* if respondents are *not* highly satisfied with the division of work. These effects are smaller when relationship satisfaction is included (M2) and finally lose statistical significance (M3).

**Table 2.** Effects of the Division of Household Labor on Childbearing Intentions by Parity (Average Marginal Effects).

Model	M1			M2			M3		
Childbearing intention	3 years	Later	None	3 years	Later	None	3 years	Later	None
Parity 0 (*N* = 2,324)									
Division of household labor									
Modernized, moderately satisfied, or less	.04	.05	−.09**	.10*	.04	−.14**	.05	.03	−.07**
Modernized, very satisfied	.04	.06*	−.10**	.06(^*^)	.06(^*^)	−.12**	.00	.06*	−.06**
Modernized, extremely satisfied	.05	.04	−.09**	.05	.04	−.09**	.00	.03	−.04(^*^)
Traditional, moderately satisfied, or less	.01	.04	−.05	.07	.03	−.10*	.00	.04	−.04
Traditional, very satisfied	.04	.00	−.04	.07	.00	−.07(^*^)	.05	−.02	−.03
Traditional, extremely satisfied (ref.)	0	0	0	0	0	0	0	0	0
Relationship satisfaction (from 0 “not satisfied” to 10 “very satisfied”)				.04***	−.01	−.03***	.02**	−.01	−.01**
Cragg and Uhler’s adjusted *R*^2^	.01			.02			.35		
Parity 1 (*N* = 3,457)									
Division of household labor									
Modernized, moderately satisfied, or less	.07(^*^)	.02	−.08*	.14***	.00	−.14***	.07*	.03	−.09**
Modernized, very satisfied	.09***	−.03	−.07*	.12***	−.03(^*^)	−.09***	.06*	−.00	−.05*
Modernized, extremely satisfied	.02	.00	−.03	.02	.01	−.03	−.01	.02	−.01
Traditional, moderately satisfied, or less	−.07*	−.01	.08*	.01	−.02	.01	.01	.00	−.01
Traditional, very satisfied	.01	.00	−.01	.03	−.01	−.03	.03	.01	−.03
Traditional, extremely satisfied (ref.)	0	0	0	0	0	0	0	0	0
Relationship satisfaction (from 0 “not satisfied” to 10 “very satisfied”)				.04***	−.01(^*^)	−.04***	.03***	−.01(^*^)	−.02***
Cragg and Uhler’s adjusted *R*^2^	.02			.03			.36		
Controls							incl.		
Constant	incl.			incl.			incl.		
Parity 2+ (*N* = 7,020)									
Division of household labor									
Modernized, moderately satisfied, or less	−.02(^*^)	−.02*	.04**	−.01	−.01	.02	−.01	−.01	.01
Modernized, very satisfied	.00	−.01	.01	.01	−.01	.00	.00	.00	.00
Modernized, extremely satisfied	.01	−.01	.00	.01	−.01	.00	.01	−.01	.00
Traditional, moderately satisfied, or less	−.03*	−.02**	.05***	−.01	−.02*	.03(^*^)	.00	−.02(^*^)	.01
Traditional, very satisfied	−.01	−.01	.02	.00	−.01	.01	.00	.00	.00
Traditional, extremely satisfied (ref.)	0	0	0	0	0	0	0	0	0
Relationship satisfaction (from 0 “not satisfied” to 10 “very satisfied”)				.01***	.00	−.01***	.01**	.00	−.01**
Cragg and Uhler’s adjusted *R*^2^	.01			.01			.14		
Controls							incl.		
Constant	incl.			incl.			incl.		

*Note.* Model M3 controls for gender, age, marriage, educational homogamy, division of professional work, economic situation, attitudes toward parenthood, and country of residence. Source: Generations and Gender Survey Wave 1 (own calculation; weights equalizing national sample sizes). Persons aged 20 to 45 years cohabiting with a partner.

(*) *p* < .1. *p < .05. **p < .01. ***p < .001.

Analyses by parity carried out separately for women and men (available as online supplemental material) indicate that the results are very similar to those presented above, although *p* values are generally higher due to lower case numbers. Basic differences between couples by parity, however, remain, and differences between women and men are once more negligible.

### The Division of Household Work and Realization of Childbearing Intentions

Descriptive results indicate that realization is somewhat higher in couples with a modernized division of household labor than in couples with a traditional division (45%-47% vs. 41%-42%) (Figure 1). The satisfaction with the division is of minor importance for realizing fertility intentions. Multivariate analyses confirm that, contrary to childbearing intentions, their realization is hardly influenced by the division of household labor (Table 3). We find some hints that couples with a modernized division of household chores who are satisfied with their division have higher probabilities of realization (M1). However, the effects do not remain statistically significant under control of other variables or in models by parity.

**Table 3.** Effects of the Division of Household Labor on Realization of Childbearing Intentions, in Total and by Parity (Average Marginal Effects).

Parity	All parities		0		1		2+	
Model	M1	M3	M1	M3	M1	M3	M1	M3
Division of household labor								
Modernized, moderately satisfied, or less	.04	.05	.01	.03	.04	.09	−.09	−.09
Modernized, very satisfied	.07*	.04	.04	.01	.03	.03	.07	.09
Modernized, extremely satisfied	.05	.01	.01	−.01	−.03	−.03	.02	.03
Traditional, moderately satisfied, or less	.02	.05	−.02	.05	−.02	−.02	.06	.04
Traditional, very satisfied	.01	.02	−.10	−.06	.06	.07	.03	.02
Traditional, extremely satisfied (ref.)	0	0	0	0	0	0	0	0
Relationship satisfaction (from 0 “not satisfied” to 10 “very satisfied”)		.03***		.04**		.03*		−.01
Stability of relationship								
Stable relationship (ref.)		0		0		0		0
Separation		−.33***		−.40***		−.30***		−.20*
Separation and new relationship		−.04		.00		−.13*		−.02
Controls		incl.		incl.		incl.		incl.
Constant	incl.	incl.	incl.	incl.	incl.	incl.	incl.	incl.
Cragg ans Uhler’s adjusted *R*^2^	.00	.19	.01	.14	.01	.22	.01	.24
*N*	2,783		1,106		1,132		545	

*Note.* Model M3 controls for gender, age, marriage, parity, educational homogamy, division of professional work, economic situation, attitudes toward parenthood, and country of residence. Source: Generations and Gender Survey Waves 1 and 2 (own calculation; weights equalizing national sample sizes). Persons aged 20 to 45 years cohabiting with a partner at Wave 1.

(*) *p* < .1. *p < .05. **p < .01. ***p < .001.

## Discussion

Scholars usually argue that a gender equal distribution of labor increases fertility. Although the empirical literature often supports this claim, observed effects of the division of family work on childbearing probabilities show substantial variation. The effects of the division of labor within couples on childbearing intentions and their realization have rarely been analyzed in detail. We developed prior research on the division of family work further in various ways, by (a) analyzing fertility intentions as well as their realization, (b) analyzing the role of the individual’s satisfaction with the division for the effects of the division on childbearing, (c) proving a mediation by relationship satisfaction, and (d) considering gender and parity as moderators.

Findings revealed that the division of work clearly influences childbearing intentions, while the division seems of less importance for their realization. Therefore, the division of work may influence birth probabilities mainly via intention building. In terms of TPB (e.g., Ajzen, 1991), the division of work is clearly confirmed as a relevant background factor in forming intentions; however, it does not appear to either be a further enabler or cause a restriction to childbearing (Hypothesis 5).

From a “gender egalitarian perspective,” a balanced division of work between spouses should strengthen childbearing intentions and foster their realization, while “cultural theories” or household economics predict higher intentions and childbearing for traditional couples. In line with the largest part of the literature, our findings mainly confirm the “gender egalitarian perspective” (e.g., Cooke, 2004; Mills et al., 2008; Oláh, 2003). Most results showed that a modernized division of work favors short-term fertility intentions. At Parity 1, however, we also found some support for a contradictory hypothesis (see below). The mechanism involved in the “gender egalitarian argument” is that a balanced division of work reduces the family burden for women, leads to higher relationship quality, and thus promotes childbearing. Our research points to at least a partial mediation of the effects of the division of labor on childbearing intentions by relationship satisfaction (Hypothesis 2). Thus, it generally supports this argument. In addition, our analyses illustrate the importance of distinguishing between the division of labor and its assessment by individuals (e.g., Coltrane, 2000; Fraser, 1994): The effect of the division is at least partly dependent on the satisfaction with the division (Hypothesis 1). Overall, our results are in line with and add to the growing body of literature arguing that the association between family work and childbearing is dependent on attitudes, prior expectations, and satisfaction regarding the division of work (e.g., Brandén, Duvander, & Ohlsson-Wijk, 2018; Goldscheider et al., 2013).

Besides mediation effects, we found evidence for moderation effects. Although associations of the division of work with childbearing intentions were stronger for women than for men (Hypothesis 3), the observed differences between women and men were small. Regarding childbearing, the interdependence of both partners on each other is obvious. An important moderator is parity (Hypothesis 4). Prior research already demonstrated that parenthood matters for the effect of an imbalanced division on women’s childbearing intentions (Mills et al., 2008). We add to the existing knowledge: *Among the childless*, a modernized division goes along with stronger fertility intentions. The gender-egalitarian argument that more equal contributions of both sexes strengthen the relationship and consequently lead to stronger fertility intentions is confirmed in this group—although only in part, as the effects remain significant under control of relationship satisfaction. The situation at Parity 1 is different. The parents already have experience with their child and are familiar with the consequences of the division of work at home. *Among parents with one child*, only those not extremely satisfied with a modernized division show above-average childbearing intentions. It seems plausible that those not satisfied with a traditional division want additional children less often, as children usually lead to a more traditional division. Intentions of respondents not satisfied with a modernized division may presumably be strongest because they would prefer more traditional lifestyles (both more children and a more traditional division of tasks between the sexes). This result may thus be in line with household economics (Becker, 1985) and preference theory (Hakim, 2003). Among parents with two or more children, intentions are stronger if parents are satisfied with the situation irrespective of whether the division is traditional or modern. We assume that those who have already reached the societal ideal and still want further children are a specific group. They want more children if they are satisfied with their present situation, including the prevalent division of household labor. In line with existing literature (e.g., Mills et al., 2008), however, further childbearing does not present an aim for an individual if the burden and strain is already perceived to be (too) large.

Our article significantly contributes to the understanding of the role of the division of family work between women and men for childbearing in contemporary European societies. Nevertheless, it has some shortcomings. First, we used merged data of four countries collected at different points in time. Sensitivity analyses, however, showed that differences between countries seem to be negligible for our main findings. A twin paper (currently in preparation) will focus on existing country differences. Second, for reasons of complexity, we could not include gender role attitudes and lifestyle preferences in our model. Future research should address interdependencies between gender role attitudes, preferences, and subjective perceptions of the division of work in analyses of childbearing. Such an approach could shed more light on the processes that shape fertility intentions and affect their realization. Third, the omission of childcare (and the satisfaction with it) may be regarded as a limitation of this article. In fact, we explored this dimension thoroughly. As childcare tasks are specifically demanding for preschool children, we focused on parents with children younger than 5 years. Overall, the division of childcare turned out to be less relevant than the division of household labor (results are available as online supplemental material).^9^ Presumably, household labor is a harder test for couples than childcare, as the latter is less monotonous and often pleasant (e.g., Gershuny, 2013; Poortman & Van der Lippe, 2009).

## Supplemental Material

Online_Supplements_(Tables) – Supplemental material for Fertility Intentions and Their Realization in Couples: How the Division of Household Chores Matters Click here for additional data file. Supplemental material, Online_Supplements_(Tables) for Fertility Intentions and Their Realization in Couples: How the Division of Household Chores Matters by Bernhard Riederer, Isabella Buber-Ennser and Zuzanna Brzozowska in Journal of Family Issues

## Notes

1. Research has at least shown that higher contributions by fathers raise both partners’ relationship satisfaction and improve relationship stability (e.g., Schober, 2012; Stevens, Kiger, & Riley, 2001).

2. A table with detailed information is available as online supplemental material. Our analyses are based on the pooled sample. Basic results on the effects of the division of work are very similar across countries (detailed results are available as online supplemental material).

3. The exact wording is, “How satisfied are you with the division of household tasks between you and your partner/spouse? Please [ . . . ] tell me the value on a scale from 0 to 10, where 0 means *not at all satisfied* and 10 means *completely satisfied*.”

4. In Austria, Hungary, and Poland, second waves were conducted 4 years after the respective first wave. We can thus identify births occurring up to 1,280 days (3 1/2 years) after the first interview. In France, the second wave was conducted after 3 years. For reasons of comparability, we thus also include pregnancies reported in second waves as births in France.

5. If others (irrespective of whether they are living in the household or not) are usually or always doing a task, we also assign “done about equally by both partners” because both partners are equal in not performing the task.

6. While women who are disadvantaged by a traditional division of household labor cannot directly influence men’s intentions of having (additional) children, they can undoubtedly impede the realization of men’s intentions.

7. Additional analyses show that the main pattern of results holds for both sexes in all four countries: A modernized division of household labor usually contributes to higher intentions, and a large part of the regression models also indicates higher intentions with higher satisfaction regarding the division (M1 in Table A.5). Due to lower case numbers, effects are, however, significant less often in models with controls (M3) for single country samples.

8. Among childless persons, 72% report a modernized and 27% a traditional division of household work (Table A.1).

9. Whereas the division of childcare turns out not to be relevant for further intentions at Parity 1, we observe lower intentions at Parity 2+ if couples are not highly satisfied with the division of childcare. Parity-specific regressions indicate that the division of childcare is not relevant for realizing fertility intentions among parents with one child, which is in line with our results on intentions. Among parents with two or more children, a modernized division of childcare raises the probabilities of realization if couples are highly satisfied with the division. In addition, probabilities of realization are higher among couples with a traditional division of childcare if they are very satisfied but not entirely satisfied with the division.
