# Supplementary material for: Fertility Intentions and Their Realization in Couples: How the Division of Household Chores Matters
Source: J Fam Issues. 2019 May 15;40(13):1860–82. doi: 10.1177/0192513X19848794 (PMC6669826; doi:10.1177/0192513X19848794)
Supplement: Online_Supplements_(Tables) – Supplemental material for Fertility Intentions and Their Realization in Couples: How the Division of Household Chores Matters [file Online_Supplements_(Tables).pdf]

Online Supplemental Material

Appendix A. Additional Tables

Table A.1: Sample selection

**Total number of records wave 1for respondents aged 20-45**		23,626
Excluded cases by cause of exclusion		
no partner	6,110	
not sharing household with partner	2,368	
no information on childbearing intentions	1,119	
(e.g., respondents physically not capable to have children,		
respondents not living in heterosexual relationship)		
no information on household labor or professional work	1,146	
other reasons (missing or contradictory information	82	
on further controls)		
Sample of analyses wave 1 (household labor)		**12,801**
Excluded cases by cause of exclusion		
childless	2,324	
parents of children above age 4	6,035	
missing information on age of youngest child	204	
missing information on division of childcare	82	
Sample of analyses wave 1 (childcare)		**4,156**
**Total number of records panel data**		33,504
Sample of analyses wave 1 (household labor) with data at wave 2		9,823
Excluded cases by cause of exclusion		
no short-term intentions	7,040	
Sample of analyses wave 2 (household labor)		**2,783**
Excluded cases by cause of exclusion		
childless	1,106	
missing information on division of childcare	129	
Sample of analyses wave 2 (childcare)		**1,548**

Source: GGS waves 1 and 2.

Table A.2: Division of family work and satisfaction at wave 1 by gender and parity

	Women	Men	Parity 0	Parity 1	Parity 2+
Division of household labor (%)					
modernized, moderately satisfied or less	10	9	12	10	9
modernized, very satisfied	18	25	28	22	17
modernized, extremely satisfied	19	30	34	25	19
traditional, moderately satisfied or less	21	5	7	13	18
traditional, very satisfied	18	14	10	15	19
traditional, extremely satisfied	14	17	10	15	17
N	7,586	5,215	2,324	3,457	7,020
Relationship satisfaction (m)					
mean on scale from 0 to 10 (sd)	8.77 (1.66)	9.10 (1.35)	9.15 (1.29)	8.93 (1.54)	8.80 (1.63)
N	7,586	5,215	2,324	3,457	7,020
Division of childcare (%)					
modernized, moderately satisfied or less	9	9		8	10
modernized, very satisfied	17	24		21	20
modernized, extremely satisfied	23	36		30	28
traditional, moderately satisfied or less	20	7		12	16
traditional, very satisfied	16	11		14	14
traditional, extremely satisfied	15	13		15	13
N	4,964	3,501		2,820	5,645

Source: GGS wave 1 (own calculation, unweighted data). Persons aged 20 to 45 years who cohabited with a partner and information to be included in multivariate regression analyses only.

Table A.3: Division of family work and satisfaction in longitudinal sample by parity

	Parity 0	Parity 1	Parity 2+	Total
Division of household labor (%)				
modernized, moderately satisfied or less	12	11	8	11
modernized, very satisfied	27	24	21	24
modernized, extremely satisfied	35	24	23	28
traditional, moderately satisfied or less	8	11	13	10
traditional, very satisfied	10	15	19	14
traditional, extremely satisfied	9	16	17	13
N	1,106	1,132	545	2,783
Relationship satisfaction (m)				
mean on scale from 0 to 10 (sd)	9.24 (1.16)	9.11 (1.30)	9.11 (1.30)	9.16 (1.25)
N	1,106	1,132	545	2,783
Division of childcare (%)				
modernized, moderately satisfied or less		7	7	7
modernized, very satisfied		21	21	21
modernized, extremely satisfied		30	28	30
traditional, moderately satisfied or less		11	14	12
traditional, very satisfied		14	16	15
traditional, extremely satisfied		17	15	16
N		1,052	496	1,548

Source: GGS wave 1 and 2 (own calculation, unweighted data). Persons aged 20 to 45 years, cohabiting with a partner, and intending to have a child within the next three years at wave 1 who participated at wave 2 and were included in longitudinal multivariate regression analyses only.

Table A.4: Additional descriptive statistics

(a) Control variables at wave 1 by gender and parity

Wave 1	Women	Men	Parity 0	Parity 1	Parity 2+
Division of professional work (%)					
at least one partner in parental leave	13	13		18	15
dual breadwinner	45	47	64	44	40
male breadwinner	18	17	12	17	20
modernized male breadwinner	12	10	6	10	14
female breadwinner/dual part-timer	8	9	12	8	8
other (e.g., no full earner)	4	4	5	3	4
Age (%)					
20-24 yrs.	8	5	20	8	1
25-34 yrs.	43	40	61	51	30
35-45 yrs.	50	56	19	41	69
Partner status (%)					
married	79	78	42	80	90
Couple's education (%)					
both partners secondary or lower	65	64	52	61	71
one partner with tertiary education	20	20	24	22	17
both partners tertiary education	15	16	24	17	12
Financial situation (%)					
bad situation	46	47	34	43	52
good situation	35	35	41	38	32
very good situation	19	18	26	19	16
Attitudes towards parenthood (%)					
children not a necessity of a fulfilled life	30	23	35	29	24
neither agreement nor disagreement	23	22	24	23	22
child(ren) necessary to lead a fulfilling life	47	55	41	48	54
N	7,586	5,215	2,324	3,457	7,020

Source: GGS wave 1 and 2 (own calculation, unweighted data). Persons aged 20 to 45 years who cohabited with a partner and information to be included in multivariate regression analyses only.

Table A.4 continued: Additional descriptive statistics

(b) Control variables in longitudinal sample

Longitudinal sample	Total	Parity 0	Parity 1	Parity 2+
Division of professional work at wave 1 (%)				
at least one partner in parental leave	16		26	26
dual breadwinner	50	70	40	32
male breadwinner	15	10	18	17
modernized male breadwinner	9	6	8	14
female breadwinner/dual part-timer	8	10	7	7
other (e.g., no full earner)	3	3	2	5
Age (%)				
20-24 yrs.	11	17	10	3
25-34 yrs.	64	69	67	50
35-45 yrs.	25	14	24	48
Partner status (%)				
married	66	46	77	83
Separation between waves (%)				
no separation (same partner at wave 1)	87	84	89	91
separation without re-partnering	4	5	4	3
separation and re-partnering (new partner)	8	11	7	7
Couple's education (%)				
both partners secondary or lower	54	48	56	64
one partner with tertiary education	24	27	22	20
both partners tertiary education	22	25	21	16
Financial situation (%)				
bad situation	39	30	41	51
good situation	39	43	38	31
very good situation	23	27	21	18
Attitudes towards parenthood (%)				
children not a necessity of a fulfilled life	26	29	26	22
neither agreement nor disagreement	22	23	22	19
child(ren) necessary to lead a fulfilling life	52	48	52	59
N	2,783	1,106	1,132	545

Source: GGS wave 1 and 2 (own calculation, unweighted data). Persons aged 20 to 45 years who cohabited with a partner and information to be included in multivariate regression analyses only. Longitudinal sample includes only those intending to have a child within the next three years at wave 1 and participating in wave 2.

Table A.5: Effects of the division of household labor on childbearing intentions by country and gender (AME)

**Women, model M1**	Austria			France			Hungary			Poland		
Childbearing intention	3 yrs	later	none	3 yrs	later	none	3 yrs	later	none	3 yrs	later	none
Division of household labor												
modernized, moderately satisfied or less	.11**	.08*	-.19***	.03	.07**	-.10	-.02	.04	-.02	.10**	.00	-.10**
modernized, very satisfied	.15***	.03	-.18***	.04	.09***	-.13*	.09**	.03	-.12***	.11***	.02	-.13***
modernized, extremely satisfied	.17***	.05*	-.22***	.03	.05*	-.07	.11**	.01	-.12**	.18***	.02	-.20***
traditional, moderately satisfied or less	.00	.00	.00	-.09	.01	.07	-.06*	-.02	.07*	-.06*	-.01	.07*
traditional, very satisfied	.06(*)	-.02	-.04	-.11(*)	.04(*)	.07	-.02	.00	.01	.01	.00	.00
traditional, extremely satisfied (ref.)	0	0	0	0	0	0	0	0	0	0	0	0
Constant	incl.			incl.			incl.			incl.		
Cragg & Uhler's adjusted R2	.05			.05			.03			.05		
**Women, model M3**	Austria			France			Hungary			Poland		
Childbearing intention	3 yrs	later	none	3 yrs	later	none	3 yrs	later	none	3 yrs	later	none
Division of household labor												
modernized, moderately satisfied or less	.04	.03	-.07(*)	-.07	.04	.03	.02	.01	-.03	.06*	-.01	-.06*
modernized, very satisfied	.08*	.00	-.08*	-.05	.05*	.00	.04	.00	-.05(*)	.02	.01	-.03
modernized, extremely satisfied	.06*	.01	-.07**	-.10(*)	.03	.07	.03	-.02	-.01	.05*	.01	-.06*
traditional, moderately satisfied or less	.04	.01	-.05	-.04	.03	.01	.02	.00	-.02	.00	.00	.00
traditional, very satisfied	.09**	-.01	-.07*	-.08	.05(*)	.03	.02	.01	-.03	.03	.00	-.03
traditional, extremely satisfied (ref.)	0	0	0	0	0	0	0	0	0	0	0	0
Controls	incl.			incl.			incl.			incl.		
Constant	incl.			incl.			incl.			incl.		
Cragg & Uhler's adjusted R2	.50			.57			.57			.59		
N	1,766			1,118			1,999			2,703		

Table A.5 continued: Effects of the division of household labor on childbearing intentions by country and gender (AME)

**Men, model M1**	Austria			France			Hungary			Poland		
Childbearing intention	3 yrs	later	none	3 yrs	later	none	3 yrs	later	none	3 yrs	later	none
Division of household labor												
modernized, moderately satisfied or less	.13(*)	-.03	-.10	.24***	.06(*)	-.29***	-.05	.01	.05	.02	.06*	-.07(*)
modernized, very satisfied	.08(*)	.02	-.10*	.21***	.05*	-.26***	.02	.02	-.04	.14***	.04**	-.18***
modernized, extremely satisfied	.09*	.00	-.09*	.20***	.05(*)	-.25***	.01	.05(*)	-.06	.10**	.05**	-.15***
traditional, moderately satisfied or less	-.31***	.05	.26**	.15(*)	.01	-.16(*)	-.08	-.05	.13*	-.11*	.03	.08
traditional, very satisfied	-.13**	-.02	.15**	.10(*)	.00	-.11(*)	.00	-.04	.04	.02	.02	-.04
traditional, extremely satisfied (ref.)	0	0	0	0	0	0	0	0	0	0	0	0
Constant	incl.			incl.			incl.			incl.		
Cragg & Uhler's adjusted R2	.06			.05			.02			.04		
**Men, model M3**	Austria			France			Hungary			Poland		
Childbearing intention	3 yrs	later	none	3 yrs	later	none	3 yrs	later	none	3 yrs	later	none
Division of household labor												
modernized, moderately satisfied or less	.11(*)	-.07(*)	-.05	.12(*)	.01	-.13(*)	.01	-.02	.01	-.01	.04(*)	-.03
modernized, very satisfied	.01	-.01	.01	.07	.01	-.08	.00	-.01	.01	-.01	.03	-.02
modernized, extremely satisfied	.00	-.03	.03	.07	.01	-.07	-.03	.01	.02	-.04	.03*	.00
traditional, moderately satisfied or less	-.36***	.18*	.18*	.11	-.03	-.08	.01	-.07(*)	.06	-.05	.03	.02
traditional, very satisfied	-.09(*)	.01	.07(*)	.14*	-.02	-.11(*)	.03	-.06*	.03	.00	.03	-.03
traditional, extremely satisfied (ref.)	0	0	0	0	0	0	0	0	0	0	0	0
Controls	incl.			incl.			incl.			incl.		
Constant	incl.			incl.			incl.			incl.		
Cragg & Uhler's adjusted R2	.50			.58			.49			.56		
N	1,037			660			1,553			1,965		

Note: Model M3 controls for gender, age, marriage, parity, educational homogamy, economic situation, and attitudes towards parenthood. (*) p < .1; * p < .05; ** p < .01; *** p < .001. Source: GGS wave 1 (own calculation; weights equalizing national sample sizes). Persons aged 20 to 45 years cohabiting with a partner.

Table A.6: Effects of the division of household labor on childbearing intentions − Testing for mediation via relationship satisfaction

	Women						Men					
**Intentions**	later vs. 3 yrs			none vs. 3 yrs			later vs. 3 yrs			none vs. 3 yrs		
Comparison of effects across models:	M1	M2	diff.	M1	M2	diff.	M1	M2	diff.	M1	M2	diff.
Division of household labor												
modernized, moderately satisfied or less	.03	-.09	.12^(*)^	-.49***	-.77***	.29***	-.04	-.33	.29***	-.29*	-.61***	.32***
modernized, very satisfied	-.06	-.11	.05^(*)^	-.65***	-.77***	.12***	-.12	-.23	.11**	-.47***	-.60***	.12**
modernized, extremely satisfied	-.17	-.17	.00	-.73***	-.74***	.00	.00	.02	-.02	-.46***	-.43***	-.03
traditional, moderately satisfied or less	-.03	-.18	.14^(*)^	.25*	-.09	.34***	.21	-.04	.25***	.47*	.20	.27***
traditional, very satisfied	.02	-.03	.05^(*)^	.01	-.11	.11***	-.21	-.33	.12**	.13	.00	.13***
traditional, extremely satisfied (ref.)												
N	7,586						5,215					

Note: Model M2 additionally includes relationship satisfaction. Tests of differences between models according to the KHB approach (Karlson, Holm, & Breen, 2012). ^(*)^ p < .1; * p < .05; ** p < .01; *** p < .001. Source: GGS wave 1 (own calculation; weights equalizing national sample sizes). Persons aged 20 to 45 years cohabiting with a partner.

Table A.7: Effects of the division of household labor on childbearing intentions by gender (AME)

Model	A1			A2			M3		
Childbearing intention	3 yrs	later	none	3 yrs	later	none	3 yrs	later	none
Women (N = 7,586)									
Division of household labor									
modernized, moderately satisfied or less	.08***	.04**	-.12***	.00	.01	-.01	.02	.01	-.03(*)
modernized, very satisfied	.11***	.04***	-.15***	.02	.01	-.04*	.03*	.01	-.05**
modernized, extremely satisfied	.14***	.03**	-.16***	.03 (*)	.00	-.03(*)	.03(*)	.00	-.03*
traditional, moderately satisfied or less	-.04*	-.01	.05**	-.01	.00	.00	.02	.00	-.02
traditional, very satisfied	.00	.00	.00	.02	.01	-.02(*)	.02	.01	-.03*
traditional, extremely satisfied (ref.)	0	0	0	0	0	0	0	0	0
Relationship satisfaction (from 0 "not satisfied" to 10 "very satisfied")							.01***	.00	-.01***
Cragg & Uhler's adjusted R^2	.05			.54			.54		
Men (N = 5,215)									
Division of household labor									
modernized, moderately satisfied or less	.06*	.02	-.09**	.00	.00	.00	.04	-.01	-.03
modernized, very satisfied	.10***	.03*	-.13***	-.01	.00	.01	.00	.00	.00
modernized, extremely satisfied	.08***	.03**	-.11***	-.01	.00	.01	-.01	.00	.01
traditional, moderately satisfied or less	-.07*	.00	.08*	-.06(*)	-.01	.07*	-.03	-.01	.04
traditional, very satisfied	-.01	-.01	.03	.00	-.02	.02	.02	-.02	.00
traditional, extremely satisfied (ref.)	0	0	0	0	0	0	0	0	0
Relationship satisfaction (from 0 "not satisfied" to 10 "very satisfied")							.03***	-.01(*)	-.02***
Cragg & Uhler's adjusted R^2	.05			.51			.51		
Controls	partly incl.			incl.			incl.		
Constant	incl.			incl.			incl.		

Note: Model A1 controls for country of residence. Models A2 and M3 control for age, marriage, parity, educational homogamy, division of professional work, economic situation, attitudes towards parenthood, and country of residence. (*) p < .1; * p < .05; ** p < .01; *** p < .001. Source: GGS wave 1 (own calculation; weights equalizing national sample sizes). Persons aged 20 to 45 years cohabiting with a partner.

Table A.8: Effects of the division of household labor on childbearing intentions by parity (AME)

Model	A1			A2			M3		
Childbearing intention	3 yrs	later	none	3 yrs	later	none	3 yrs	later	none
Parity 0 (N = 2,324)									
Division of household labor									
modernized, moderately satisfied or less	.04	.08*	-.13***	.02	.03	-.05(*)	.05	.03	-.07**
modernized, very satisfied	.04	.09***	-.13***	-.01	.07*	-.06*	.00	.06*	-.06**
modernized, extremely satisfied	.05	.06*	-.11***	.01	.03	-.04	.00	.03	-.04(*)
traditional, moderately satisfied or less	.01	.05	-.06	-.02	.04	-.02	.00	.04	-.04
traditional, very satisfied	.04	.01	-.05	.04	-.01	-.04	.05	-.02	-.03
traditional, extremely satisfied (ref.)	0	0	0	0	0	0	0	0	0
Relationship satisfaction (from 0 “not satisfied” to 10 “very satisfied”)							.02**	-.01	-.01**
Cragg & Uhler's adjusted R^2	.08			.35			.35		
Parity 1 (N = 3,457)									
Division of household labor									
modernized, moderately satisfied or less	.03	.04(*)	-.08*	.05	.03	-.08*	.07*	.03	-.09**
modernized, very satisfied	.07*	.00	-.06*	.05(*)	.00	-.05*	.06*	-.00	-.05*
modernized, extremely satisfied	.01	.02	-.03	-.00	.02	-.02	-.01	.02	-.01
traditional, moderately satisfied or less	-.09**	.01	.08**	-.02	.01	.02	.01	.00	-.01
traditional, very satisfied	.00	.00	.00	.01	.01	-.02	.03	.01	-.03
traditional, extremely satisfied (ref.)	0	0	0	0	0	0	0	0	0
Relationship satisfaction (from 0 “not satisfied” to 10 “very satisfied”)							.03***	-.01(*)	-.02***
Cragg & Uhler's adjusted R^2	.06			.38			.36		
Controls	partly incl.			incl.			incl.		
Constant	incl.			incl.			incl.		

Table A8 continued: Effects of the division of household labor on childbearing intentions by parity (AME)

Model	A1			A2			M3		
Childbearing intention	3 yrs	later	none	3 yrs	later	none	3 yrs	later	None
Parity 2+ (N = 7,020)									
Division of household labor									
modernized, moderately satisfied or less	-.02	-.02(*)	.04*	-.02	-.01	.03	-.01	-.01	.01
modernized, very satisfied	.00	-.01	.01	.00	.00	.00	.00	.00	.00
modernized, extremely satisfied	.01	-.01	.00	.02	.00	-.01	.01	-.01	.00
traditional, moderately satisfied or less	-.03*	-.02**	.05***	-.02	-.02*	.03*	.00	-.02(*)	.01
traditional, very satisfied	-.01	-.01	.02	-.01	.00	.01	.00	.00	.00
traditional, extremely satisfied (ref.)	0	0	0				0	0	0
Relationship satisfaction (from 0 “not satisfied” to 10 “very satisfied”)							.01**	.00	-.01**
Cragg & Uhler's adjusted R^2	.01			.14			.14		
Controls	partly incl.			incl.			incl.		
Constant	incl.			incl.			incl.		

Note: Model A1 controls for country of residence. Models A2 and M3 control for gender, age, marriage, educational homogamy, division of professional work, economic situation, attitudes towards parenthood, and country of residence. (*) p < .1; * p < .05; ** p < .01; *** p < .001. Source: GGS wave 1 (own calculation; weights equalizing national sample sizes). Persons aged 20 to 45 years cohabiting with a partner.

Table A.9: Effects of the division of household labor on childbearing intentions, additional tests for mediation via relationship satisfaction

**Difference in coefficients (none vs. 3 yrs) between by**	Models A1 and A1+					Models A2 and M3				
	Gender		Parity			Gender		Parity		
	women	men	0	1	2+	women	men	0	1	2+
Division of household labor										
modernized, moderately satisfied or less	.29***	.30***	.38***	.31***	.21**	.20***	.27***	.23*	.25**	.17*
modernized, very satisfied	.12***	.11*	.16(*)	.13*	.08**	.08*	.10(*)	.10	.11	.06
modernized, extremely satisfied	.00	-.02	.00	.01	-.01	.01	-.02	.01	.01	-.01
traditional, moderately satisfied or less	.34***	.25***	.38***	.35***	.22**	.22***	.22**	.23*	.27**	.19*
traditional, very satisfied	.11***	.12**	.17*	.12*	.07*	.07(*)	.11(*)	.10	.10	.06
traditional, extremely satisfied (ref.)										
N	7,586	5,215	2,324	3,457	7,020	7,586	5,215	2,324	3,457	7,020

Note: Model A1 controls for country of residence. Model A1+ additionally includes relationship satisfaction. Models A2 and M3 control for gender, age, marriage, parity, educational homogamy, division of professional work, economic situation, attitudes towards parenthood, and country of residence. Model M3 additionally includes relationship satisfaction. Tests of differences between models according to the KHB approach (Karlson, Holm, & Breen, 2012; see also Table A.6).
(*) p < .1; * p < .05; ** p < .01; *** p < .001. Source: GGS wave 1 (own calculation; weights equalizing national sample sizes). Persons aged 20 to 45 years cohabiting with a partner.

Table A.10: Effects of the division of household labor on childbearing intentions by parity and gender (AME)

Parity 0	Women (N = 1,261)						Men (N = 1,063)					
Model	M1			M3			M1			M3		
Childbearing intention	3 yrs	later	none	3 yrs	later	none	3 yrs	later	none	3 yrs	later	none
Division of household labor												
modernized, moderately satisfied or less	.05	.09*	-.14**	-.01	.08	-.08*	.04	.00	-.04	.13*	-.04	-.08(*)
modernized, very satisfied	.05	.11**	-.16***	-.04	.12**	-.08**	.03	-.01	-.02	.04	.00	-.05
modernized, extremely satisfied	.07	.07*	-.14***	-.02	.07	-.05(*)	.02	.00	-.02	.03	.00	-.03
traditional, moderately satisfied or less	.04	.07	-.11*	-.04	.10(*)	-.06	-.22(*)	.10	.11	-.07	.02	.05
traditional, very satisfied	.01	.06	-.08	-.03	.08	-.05	.08	-.07	-.01	.15*	-.13*	-.02
traditional, extremely satisfied (ref.)	0	0	0	0	0	0	0	0	0	0	0	0
Relationship satisfaction (from 0 “not satisfied” to 10 “very satisfied”)				.01	.00	-.01(*)				.03**	-.02*	-.01(*)
Controls				incl.						incl.		
Constant	incl.			incl.			incl.			incl.		
Cragg & Uhler's adjusted R2	.02			.40			.01			.33		

Table A.10 continued: Effects of the division of household labor on childbearing intentions by parity and gender (AME)

Parity 1	Women (N = 1,975)						Men (N = 1,482)					
Model	M1			M3			M1			M3		
Childbearing intention	3 yrs	later	none	3 yrs	later	none	3 yrs	later	none	3 yrs	later	none
Division of household labor												
modernized, moderately satisfied or less	.06	.01	-.06	.07(*)	.02	-.10*	.09	.02	-.11*	.07	.02	-.10*
modernized, very satisfied	.10**	-.06*	-.05	.08*	-.03	-.04	.08*	.01	-.09*	.04	.03	-.08*
modernized, extremely satisfied	.04	-.03	-.01	.02	-.01	-.01	.00	.05(*)	-.05	-.03	.06*	-.02
traditional, moderately satisfied or less	-.07(*)	-.03	.10**	.01	-.01	.00	.02	.01	-.02	.00	.01	-.01
traditional, very satisfied	-.01	-.02	.03	.03	-.01	-.02	.05	.02	-.07	.04	.03	-.07(*)
traditional, extremely satisfied (ref.)				0	0	0	0	0	0	0	0	0
Relationship satisfaction (from 0 “not satisfied” to 10 “very satisfied”)				.03***	-.01	-.02**				.04***	-.01	-.03***
Controls				incl.						incl.		
Constant	incl.			incl.			incl.			incl.		
Cragg & Uhler's adjusted R2	.02			.41			.01			.32		

Table A.10 continued: Effects of the division of household labor on childbearing intentions by parity and gender (AME)

Parity 2+	Women (N = 4,350)						Men (N = 2,670)					
Model		M1			M3			M1			M3	
Childbearing intention	3 yrs	later	none	3 yrs	later	none	3 yrs	later	none	3 yrs	later	none
Division of household labor												
modernized, moderately satisfied or less	-.02	-.02*	.04$^{(*)}$	.00	-.02	.02	-.03	-.02	.05$^{(*)}$	.00	.00	.01
modernized, very satisfied	.02	.00	-.02	.03$^{(*)}$	.00	-.03$^{(*)}$	-.03	-.02$^{(*)}$	.06*	-.02	-.01	.03
modernized, extremely satisfied	.02	.00	-.02	.03$^{(*)}$	.00	-.03$^{(*)}$	-.01	-.02$^{(*)}$	.04	-.01	-.02	.02
traditional, moderately satisfied or less	-.01	-.02*	.02	.02	-.01	-.01	-.06*	-.02	.08*	-.04	-.02	.06$^{(*)}$
traditional, very satisfied	.02	.00	-.02	.02$^{(*)}$	.01	-.03$^{(*)}$	-.05*	-.03$^{(*)}$	.07***	-.03	-.02	.04$^{(*)}$
traditional, extremely satisfied (ref.)	0	0	0	0	0	0				0	0	0
Relationship satisfaction (from 0 “not satisfied” to 10 “very satisfied”)				.01*	.00	-.01*				.01*	.00	-.02*
Controls					incl.						incl.	
Constant		incl.			incl.			incl.			incl.	
Cragg & Uhler's adjusted R2		.01			.15			.01			.15	

Note: Model M3 controls for age, marriage, educational homogamy, economic situation, attitudes towards parenthood, and country of residence. $^{(*)}$ $p < .1$; * $p < .05$; ** $p < .01$; *** $p < .001$. Source: GGS wave 1 (own calculation; weights equalizing national sample sizes). Persons aged 20 to 45 years cohabiting with a partner.

Table A.11: Effects of the division of household labor and childcare on childbearing intentions of parents (AME)

Parents	Parity 1 (N = 3,457)						Parity 2+ (N = 7,020)					
Model	M1			M3			M1			M3		
Childbearing intention	3 yrs	later	none	3 yrs	later	none	3 yrs	later	none	3 yrs	later	none
Division of household labor												
modernized, moderately satisfied or less	.10**	.02	-.12***	.08*	.03	-.10***	-.02	-.01	.03	-.01	.00	.01
modernized, very satisfied	.11***	-.02	-.09**	.06*	.00	-.06*	.00	.00	.00	.00	.00	.00
modernized, extremely satisfied	.04	.01	-.05(*)	.00	.02	-.02	.02	-.01	-.01	.02	.00	-.01
traditional, moderately satisfied or less	-.03	-.09	.04	.01	.00	-.01	-.02(*)	-.02*	.04**	-.01	-.01(*)	.02
traditional, very satisfied	.02	.00	-.02	.02	.01	-.03	-.01	.00	.02	.00	.00	.00
traditional, extremely satisfied (ref.)	0	0	0	0	0	0	0	0	0	0	0	0
Division of childcare												
modernized, moderately satisfied or less	-.07	.00	.07(*)	.00	.01	-.02	-.01	-.04***	.05*	.01	-.02*	.01
modernized, very satisfied	-.07*	-.02	.09**	-.03	-.01	.04	-.01	-.02*	.03(*)	.00	-.01	.01
modernized, extremely satisfied	-.07*	-.03	.09***	-.02	-.01	.03	-.02	-.02(*)	.04*	-.01	-.01	.02
traditional, moderately satisfied or less	-.08*	.00	.08*	-.02	.00	.02	-.01	.00	.01	.01	.00	-.01
traditional, very satisfied	-.03	-.02	.05	.02	-.01	-.01	.02	-.01	-.01	.02	-.01	-.01
traditional, extremely satisfied (ref.)	0	0	0	0	0	0	0	0	0	0	0	0
Division of professional work				incl.						incl.		
Relationship satisfaction				incl.						incl.		
Controls				incl.						incl.		
Constant	incl.			incl.			incl.			incl.		
Cragg & Uhler's adjusted R2	.16			.38			.03			.14		

Note: All models additionally include a dummy variable indicating missing information on childcare (information is missing if the youngest child is 14 years old or older). Model M3 controls for age, marriage, educational homogamy, economic situation, attitudes towards parenthood, and country of residence. (*) $p < .1$; * $p < .05$; ** $p < .01$; *** $p < .001$. Source: GGS wave 1 (own calculation; weights equalizing national sample sizes). Persons aged 20 to 45 years cohabiting with a partner.

Table A.12: Effects of the division of childcare on childbearing intentions of parents of children below 5 by parity (AME)

Parents of children below 5	Parity 1 (N = 1,688)						Parity 2+ (N = 2,468)					
Model	M1			M3			M1			M3		
Childbearing intention	3 yrs	later	none	3 yrs	later	none	3 yrs	later	none	3 yrs	later	none
Division of childcare												
modernized, moderately satisfied or less	-.03	-.01	.04	-.02	.01	.01	-.04	-.06***	.10**	.01	-.07*	.06
modernized, very satisfied	.02	-.04	.02	.00	-.01	.01	-.02	-.02	.04	.00	-.02	.02
modernized, extremely satisfied	.01	-.03	.01	.01	.00	-.01	-.02	.02	.04	-.03	-.02	.04
traditional, moderately satisfied or less	.00	-.03	.03	.03	-.03	.00	-.06*	-.03	.09**	-.01	-.01	.02
traditional, very satisfied	-.02	-.02	.04	-.01	.00	.01	.00	-.02	.02	.02	-.02	.00
traditional, extremely satisfied (ref.)	0	0	0	0	0	0	0	0	0	0	0	0
Division of household labor				incl.						incl.		
Division of professional work				incl.						incl.		
Relationship satisfaction				incl.						incl.		
Controls				incl.						incl.		
Constant	incl.			incl.			incl.			incl.		
Cragg & Uhler's adjusted R2	.00			.17			.01			.13		

Note: Model M3 controls for gender, age, marriage, educational homogamy, economic situation, attitudes towards parenthood, and country of residence. (*) p < .1; * p < .05; ** p < .01; *** p < .001. Source: GGS wave 1 (own calculation; weights equalizing national sample sizes). Persons aged 20 to 45 years cohabiting with a partner.

Table A.13: Effects of the division of childcare on realization of childbearing intentions, in total and by parity (AME)

Parity	1		2+		all parities		
Model	M1	M3	M1	M3	M1	M1+	M3+
Division of childcare							
modernized, moderately satisfied or less	-.01	.01	.03	.12	.02	.05	.05
modernized, very satisfied	-.02	.02	.07	.10	.01	.05	.07
modernized, extremely satisfied	-.02	.04	.06	.13*	.00	.04	.08*
traditional, moderately satisfied or less	.01	.07	-.03	-.03	-.03	.01	.04
traditional, very satisfied	.05	.06	.21**	.16*	.11*	.13**	.10*
traditional, extremely satisfied (ref.)	0	0	0	0	0	0	0
Division of household labor		incl.		incl.			incl.
Division of professional work		incl.		incl.			incl.
Relationship satisfaction		incl.		incl.			incl.
Controls		incl.		incl.			incl.
Constant	incl.	incl.	incl.	incl.	incl.	incl.	incl.
Cragg & Uhler's adjusted R2	.00	.20	.04	.26	.01	.11	.22
N	1,052		496		1,548		

Note: Model M3 controls for gender, age, marriage, educational homogamy, economic situation, attitudes towards parenthood, and country of residence. Models M1+ and M3+ (additionally) control for age of youngest child. (*) $p < .1$; * $p < .05$; ** $p < .01$; *** $p < .001$. Source: GGS waves 1 and 2 (own calculation; weights equalizing national sample sizes). Parents aged 20 to 45 years cohabiting with a partner at wave 1.

Appendix B. Measurement of Childcare and Controls

As with household labor, a stepwise approach is applied to account for the *division of childcare* (details are described in the main text). Both the division of childcare tasks itself and the satisfaction with the division of childcare are considered. The six chores involved in index construction comprise dressing the children, putting them to bed, staying at home when they are ill, playing/ taking part in leisure activities with them, helping with homework, and taking them to/ from school, daycare, babysitter or leisure activities ($\alpha = .80$). We finally distinguish between six combinations of the division of work (modernized/ traditional) and the satisfaction with this division (extremely satisfied/ very satisfied/ moderately or less satisfied).

As *control variables* we consider: (a) gender, (b) age, (c) partner status at wave 1, (d) separation and repartnering between waves (only in longitudinal analyses), (e) couples' education, (f) division of professional work, (g) financial situation, (h) attitudes towards parenthood, (i) parity, and (j) country of residence. Analyses for the division of childcare further account for (k) the age of youngest child.

Age is considered in three categories: 20-24, 25-34, and 35-45 years. *Partner status* at wave 1 differentiates between married and cohabiting couples. Addressing *separation of the couple and repartnering* between wave 1 and wave 2, we consider the categories no separation, separation and single at wave 2, and separation and new partner at wave 2. This item is used only in longitudinal analyses. *Couples' education* is assessed in three categories as follows: both partners with non-tertiary education, one partner with tertiary and the other one with non-tertiary education, and both with tertiary education.

The *division of professional work* of a couple is captured by means of a typology. Following the scheme suggested by Steiber, Berghammer, and Haas (2016), we distinguish between: (1) male breadwinner couples (i.e. male partner works at least 30 hours per week, female partner not employed), (2) couples with at least one partner in parental leave, (3) dual breadwinner couples (each partner works at least 30 hours per week), (4) modernized male breadwinner couples (male partner works at least 30 hours per week while female partner works less than 30 hours per week), (5) female breadwinner/ dual part-timer couples, and (6) couples with no full earner.

Financial situation is measured using self-assessments of perceived economic constraints. Respondents were asked “Thinking of your household’s total monthly income, is your household able to make ends meet?” Possible answers were (1) with great difficulty, (2) with difficulty, (3) with some difficulty, (4) fairly easily, (5) easily and (6) very easily. We collapse the scheme to differentiate between a bad economic situation (values between 1 and 3), a good situation (value 4) and a very good economic situation (values 5 and 6).

Attitudes towards parenthood is measured in terms of agreement towards the statements “A woman has to have children in order to be fulfilled” and “A man has to have children in order to be fulfilled”. Possible answers range from (1) “Strongly agree”, (2) “agree”, to (3) “neither agree nor disagree”, (4) “disagree” and (5) “strongly disagree”. We use the average rating on both items (or the only rating available in case that respondents did not answer on both of them). Answers are collapsed into three categories as follows: Values between 1.0 and 2.5 indicate agreement to the statement that a child is necessary to lead a fulfilling life and values between 4.0 and 5.0 stand for disagreement to this statement (meaning that children are not perceived as necessity of a fulfilled life). Values in-between (i.e. 3.0 and 3.5) are interpreted neither as agreement nor as disagreement.

Regarding *parity*, we differentiate between childless respondents, parents of one child, and parent of two or more children. *Country of residence* indicates whether participants live in Austria, France, Hungary, or Poland. The *age of youngest child* is considered in three categories: below 3 years, 3-5 years, and 6 years and older.

References

Karlson, K. B., Holm, A., & Breen, R. (2012). Comparing regression coefficients between same-sample nested models using logit and probit a new method. *Sociological Methodology, 42*(1), 286-313.

Steiber, N., Berghammer, C., & Haas, B. (2016). Contextualizing the education effect on women's employment: a cross-national comparative analysis. *Journal of Marriage and Family, 78*(1), S. 246-261. doi: 10.1111/jomf.12256
